# Outcomes and risk factors of COVID-19 in patients with bullous pemphigoid: a cross-sectional study

**DOI:** 10.3389/fimmu.2025.1568801

**Published:** 2025-04-25

**Authors:** Yuexin Zhang, Dawei Huang, Yuling Shi, Yangfeng Ding, Yunlu Gao

**Affiliations:** ^1^ Department of Dermatology, Shanghai Skin Disease Hospital, Tongji University School of Medicine, Shanghai, China; ^2^ Institute of Psoriasis, Tongji University School of Medicine, Shanghai, China

**Keywords:** bullous pemphigoid, COVID-19, SARS-CoV-2, glucocorticoids, vaccination

## Abstract

**Background:**

The outcomes of coronavirus disease 2019 (COVID-19) in patients with bullous pemphigoid (BP) remain insufficiently understood. This study aimed to evaluate the impact of COVID-19 on BP patients and identify factors influencing the risk and severity of severe acute respiratory syndrome coronavirus 2 (SARS-CoV-2) infection in BP patients.

**Methods:**

A cross-sectional survey was conducted among BP patients in the Department of Dermatology at Shanghai Skin Disease Hospital from December 1, 2022, to March 1, 2023. Participants completed a comprehensive questionnaire addressing demographics, medical conditions, clinical symptoms, and behaviors during the COVID-19 pandemic. Factors influencing the risk of SARS-CoV-2 infection and its severity were evaluated by logistic regression.

**Results:**

A total of 96 BP patients were analyzed. Demographic and clinical profiles, COVID-19-related characteristics, and the pandemic’s impact on healthcare-seeking behaviors were described. Our findings showed that vaccination was associated with a reduced risk of SARS-CoV-2 infection (odds ratio [OR]: 0.157, 95% confidence interval [CI]: 0.045–0.552, p=0.002) and infection severity (OR: 0.044, 95% CI: 0.004–0.544, p=0.015). Furthermore, the use of prednisone at a dose >10 mg/day in the last 3 months was associated with an increased risk of SARS-CoV-2 infection (OR: 7.911, 95% CI: 1.379–45.393, p=0.012) but did not appear to influence infection severity.

**Conclusions:**

The COVID-19 pandemic posed significant challenges for BP patients. Our study found that the use of prednisone at a dose >10 mg/day in the last three months was associated with an increased risk of SARS-CoV-2 infection. Vaccination provided protection against SARS-CoV-2 infection and severe COVID-19 in BP patients.

## 1 Introduction

Bullous pemphigoid (BP) is the most common subepidermal autoimmune bullous disease (AIBD) (1). Prolonged immunosuppression from treatment, combined with disruptions to the skin barrier, increases the susceptibility of BP patients to infections. Infections remain a leading cause of mortality in BP patients (2, 3).

At the end of 2019, coronavirus disease 2019 (COVID-19), caused by severe acute respiratory syndrome coronavirus 2 (SARS-CoV-2), spread across the globe. Concerns have been raised regarding the impact of COVID-19 on BP patients. During the pandemic, maladaptive healthcare behaviors, such as delaying medical consultations or self-adjusting prescribed treatments, likely disrupted BP management. BP patients, particularly those undergoing immunosuppressive therapy, may face an elevated risk of severe infections and have higher mortality rates (4). Research on COVID-19 outcomes in BP patients remains limited, and existing studies have reported conflicting results, highlighting the need for further investigation. With the potential for recurring waves of the pandemic, this issue remains relevant.

This study aimed to assess the impact of COVID-19 on BP patients and to identify factors influencing the risk and severity of SARS-CoV-2 infection among individuals with BP.

## 2 Methods

### 2.1 Study design and subjects

This single-center cross-sectional study was conducted at the Department of Dermatology, Shanghai Skin Disease Hospital, Shanghai, China, from December 1, 2022, to March 1, 2023. Patients diagnosed with BP who regularly attended follow-up appointments at our outpatient clinic were included. Data was collected using a real-name questionnaire. The study received ethical approval from the Shanghai Skin Disease Hospital ethics committee and adhered to the principles of the Declaration of Helsinki. All participants provided informed consent for data collection and publication.

The questionnaire comprised multiple sections, addressing participants’ demographics, clinical symptoms, medical conditions, and behaviors during the COVID-19 pandemic. The sections included:

- General patient information: Age, gender, and comorbidities.
- BP status during COVID-19: Duration of BP and treatment regimens.
- COVID-19 status: Symptoms, disease severity, and treatments received.
- Medical behaviors during COVID-19: Vaccination status, healthcare-seeking behaviors, adherence to follow-up visits, and use of telemedicine services.

### 2.2 BP and COVID-19 identification

BP diagnosis was confirmed based on clinical manifestations, histopathological examination, immunopathological assessment, and enzyme-linked immunosorbent assay (ELISA), following the diagnostic criteria established by the European Academy of Dermatology and Venereology (1). Patients with incomplete questionnaires were excluded.

COVID-19 diagnosis was confirmed through positive nucleic acid amplification tests (NAAT), or by meeting clinical and/or epidemiological criteria in conjunction with a positive result from either a professional-use or self-test SARS-CoV-2 antigen detection rapid diagnostic test (Ag-RDT). The severity of COVID-19 was classified according to previously reported (5, 6): mild (including symptoms such as cough, fever, fatigue, change in taste or smell, and no dyspnea); moderate (with clinical or radiographic evidence of lower respiratory tract disease, oxygen saturation >94%); severe (with oxygen saturation <94%, respiratory rate >30 breaths/min, lung infiltrates >50%); and critical (involving respiratory failure, shock, and multiorgan dysfunction or failure).

### 2.3 Statistical analysis

Quantitative data with a normal distribution were presented as mean ± standard deviation (SD), and group differences were analyzed using Student’s t-test. Nonnormally distributed data were expressed as the median and interquartile range (IQR) and analyzed using the Mann–Whitney U test. Qualitative data were presented as frequencies and percentages, with comparisons conducted using Pearson’s chi-square test or Fisher’s exact test, as appropriate. Binary logistic regression models were employed to identify factors contributing to the risk of SARS-CoV-2 infection. Ordinal logistic regression models were applied to assess factors influencing the severity of SARS-CoV-2 infection. A two-sided P value <0.05 was considered statistically significant. All data were analyzed using IBM SPSS Statistics software (SPSS Inc, Chicago, IL, USA, version 26.0).

## 3 Results

### 3.1 Demographic and clinical profile of the study population

A total of 100 questionnaires were collected, with 4 incomplete responses excluded, resulting in a final cohort of 96 patients with BP. All enrolled patients were in the remission phase of BP. **Table 1** summarizes the demographic and clinical profiles of BP patients with and without SARS-CoV-2 infection. Among them, 41 (42.7%) were women, and 55 (57.3%) were men. The mean age of the cohort was 72.9 years (SD=11.4), ranging from 43 to 94 years. The median BP duration was 51.0 weeks (IQR: 18.5–110.8). Hypertension was the most prevalent comorbidity, followed by type 2 diabetes mellitus, cardiac disease and so on. Most participants reported no respiratory comorbidities, while a minority indicated conditions such as chronic bronchitis, asthma or pulmonary embolism. Regarding treatment, systemic corticosteroids were the most frequently prescribed, with additional therapies including dupilumab, methotrexate, doxycycline, Janus kinase inhibitors, and topical corticosteroids.

**Table 1:** Demographics and baseline characteristics of BP patients.

	Over all N = 96	Covid-19 (+) N = 75	Covid-19 (-) N = 21	p-value
Age (year), Mean ± SD	72.9 ± 11.4	71.7 ± 11.9	74.5 ± 7.4	0.185^T^
Gender				0.988^X^
Male	55 (57.3%)	43 (57.3%)	12 (57.1%)	
Female	41 (42.7%)	32 (42.7%)	9 (42.9%)	
BP duration (weeks), Median (IQR)	51.0 (18.5, 110.8)	51.0 (17.0, 112.0)	51.0 (40.0, 118.0)	0.520^M^
COVID-19 vaccination				0.002^X^
Vaccinated	40 (41.7%)	25 (33.3%)	15 (71.4%)	
Non-vaccinated	56 (58.3%)	50 (66.7%)	6 (28.6%)	
Comorbidities				/
Hypertension	53 (55.2%)	43 (57.3%)	10 (47.6%)	
T2DM	33 (34.4%)	27 (36.0%)	6 (28.6%)	
Cardiac disease	22 (22.9%)	18 (24.0%)	4 (19.0%)	
Cerebrovascular disease	17 (17.7%)	13 (17.3%)	4 (19.0%)	
Chronic bronchitis	9 (9.4%)	8 (10.7%)	1 (4.8%)	
Malignancy	8 (8.3%)	4 (5.3%)	4 (19.0%)	
Hepatitis B	6 (6.3%)	5 (6.7%)	1 (4.8%)	
Psoriasis	6 (6.3%)	4 (5.3%)	2 (9.5%)	
Alzheimer’s disease	4 (4.2%)	3 (4.0%)	1 (4.8%)	
Asthma	3 (3.1%)	3 (4.0%)	0 (0%)	
Parkinson’s disease	3 (3.1%)	2 (2.7%)	1 (4.8%)	
Osteoporosis	3 (3.1%)	3 (4.0%)	0 (0%)	
Pulmonary embolism	1 (1.0%)	1 (1.3%)	0 (0%)	
ONFH	1 (1.0%)	1 (1.3%)	0 (0%)	
Treatment				/
SCS	70 (72.9%)	50 (66.7%)	20 (95.2%)	
DOX	1 (1.0%)	1 (1.3%)	0 (0.0%)	
DUP	4 (4.2%)	4 (5.3%)	0 (0.0%)	
JAKi	2 (2.1%)	2 (2.7%)	0 (0.0%)	
TCS	2 (2.1%)	2 (2.7%)	0 (0.0%)	
SCS+DOX	3 (3.1%)	3 (4.0%)	0 (0.0%)	
SCS+MTX	7 (7.3%)	7 (9.3%)	0 (0.0%)	
SCS+DUP	5 (5.2%)	4 (5.3%)	1 (4.8%)	
SCS+JAKi	2 (2.1%)	2 (2.7%)	0 (0.0%)	
Prednisone dose in the last 3 months				0.023^F^
>10 mg/d	21 (30.0%)	19 (38.0%)	2 (10.0%)	
≤10 mg/d	49 (70.0%)	31 (62.0%)	18 (90.0%)	

^T^Student’s T test; ^X^Chi-square test; ^M^Mann Whitney U test; ^F^Fisher’s Exact test.

BP, bullous pemphigoid; SD, standard deviation; IQR, interquartile range; COVID-19, coronavirus disease 2019; T2DM, diabetes mellitus type 2; ONFH, osteonecrosis of the femoral head; SCS, systemic corticosteroids; DOX, doxycycline; DUP, dupilumab; JAKi, Janus kinases inhibitor; TCS, topical corticosteroids; MTX, methotrexate; mg/d, mg/day.

Among the 96 patients, 40 (41.7%) were fully vaccinated with the COVID-19 vaccine (Sinovac Biotech) before exposure, 25 of whom subsequently contracted COVID-19. Reasons for not receiving the vaccine included concerns about exacerbation or recurrence of BP (31 patients), underlying health conditions (10 patients), and advanced age (12 patients). Some individuals cited multiple reasons for avoiding vaccination. Among the 40 vaccinated patients, 6 experienced recurrence or exacerbation of BP. Specifically, 5 patients reported recurrence after the second vaccine dose, while 1 patient experienced recurrence following the first dose.

### 3.2 COVID-19-related characteristics of the cohort

A total of 75 patients (78.1%) with BP tested positive for COVID-19. **Table 2** summarizes the COVID-19-related characteristics of these SARS-CoV-2-positive patients. The severity of COVID-19 among these patients was categorized as follows: asymptomatic in 6 patients (8.0%), mild in 55 patients (73.3%), moderate in 11 patients (14.7%), severe in 2 patients (2.7%), and critical in 1 patient (1.3%). The patient in whom the severity of COVID-19 was critical succumbed to the illness. The most commonly reported symptoms among the patients were fever, cough, fatigue, and muscle soreness. COVID-19 management strategies varied among patients, with traditional Chinese medicine and antipyretics being the most frequently used treatments.

**Table 2:** COVID-19-related characteristics of SARS-CoV-2 infected BP patients.

COVID-19-related characteristics	Number (percentage)
COVID-19 severity	
Mild	55 (73.3%)
Moderate	11 (14.7%)
Severe	2 (2.7%)
Critical	1 (1.3%)
Asymptomatic	6 (8.0%)
COVID-19-related symptoms	
Fever	45 (60.0%)
Cough	44 (58.7%)
Fatigue	41 (54.7%)
Muscle soreness	41 (54.7%)
Expectoration	25 (33.3%)
Pharyngalgia	21 (28.0%)
Headache	18 (24.0%)
Catarrhal symptoms	9 (12.0%)
Dyspnea	4 (5.3%)
Increased heart rate	4 (5.3%)
Decreased blood oxygen saturation	3 (4.0%)
Shortness of breath	3 (4.0%)
Abnormal taste or smell	2 (2.7%)
COVID-19 management strategies	
Traditional Chinese medicine	38 (50.7%)
Antipyretics	36 (48.0%)
Cough expectorants	10 (13.3%)
Antibiotics	7 (9.3%)
Antiviral drugs	3 (4.0%)
Intravenous immunoglobulin G	1 (1.3%)
No medical treatment for COVID-19	21 (28.0%)

COVID-19, coronavirus disease 2019; SARS-CoV-2, acute respiratory syndrome coronavirus 2; BP, bullous pemphigoid.

### 3.3 The impact of the COVID-19 pandemic on BP patients and their healthcare-seeking behaviors

A total of 32 patients exhibited negative health behaviors during the COVID-19 pandemic. These behaviors included fear and avoidance of follow-up hospital visits (21 patients, 65.63%), reduction of medication dosage (9 patients, 28.13%), and discontinuation of medication without medical consultation (2 patients, 6.26%). Among the 11 patients who self-reduced or discontinued BP medication, 5 believed it was appropriate to taper off treatment, 3 were concerned that glucocorticoids and immunosuppressants might compromise immunity and interfere with COVID-19 recovery, 2 feared potential drug interactions, and 1 independently discontinued medication following a sudden syncope episode.

Regarding follow-up care, five patients experienced delays in treatment. However, most patients avoided treatment delays thanks to the increased use of telemedicine. Thirty-eight patients utilized telemedicine services, including WeChat groups (10 patients), official hospital online clinics (23 patients), and other online platforms (5 patients). Reasons for not using online follow-up included difficulty operating smartphones (14 patients), lack of awareness of these services (11 patients), and others.

Of the 96 patients, 8 (8.3%) experienced BP recurrence following SARS-CoV-2 infection, necessitating additional or adjusted treatment. None of these patients had independently discontinued or reduced their BP medication before the recurrence. Two of these patients used a web-based platform for follow-up, while one patient tragically passed away due to complications related to COVID-19 and BP.

### 3.4 Risk and severity of SARS-CoV-2 infection among patients with BP

**Figure 1:**
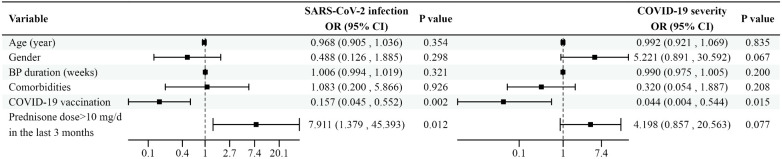
Forest plot depicting the impact of potential risk factors on SARS-CoV-2 infection and disease severity in BP patients. Variables with the corresponding odds ratios and confidence intervals are shown in the figure.SARS-CoV-2, acute respiratory syndrome coronavirus 2; BP, bullous pemphigoid; COVID-19, coronavirus disease 2019; OR, odds ratio; CI, confidence interval; mg/d, mg/day.

A summary of risk factors associated with SARS-CoV-2 infection and disease severity is presented in **Figure 1**.

Patients were categorized based on their COVID-19 status, and a binary logistic regression model was used to identify risk factors for SARS-CoV-2 infection in BP patients. Variables included in the analysis were age (years), gender, BP duration (weeks), comorbidities, COVID-19 vaccination status, and whether the use of prednisone at a dose >10 mg/day (mg/d) in the last 3 months. The analysis revealed that vaccination was associated with a reduced odds of SARS-CoV-2 infection (odds ratio [OR]: 0.157, 95% confidence interval [CI]: 0.045–0.552, p=0.002). Conversely, the use of prednisone at a dose >10 mg/d in the last 3 months was associated with an increased risk of SARS-CoV-2 infection in BP patients (OR: 7.911, 95% CI: 1.379–45.393, p=0.012). No significant independent effects were observed for age, gender, or comorbidities.

For COVID-19-positive patients, the severity of infection was categorized as asymptomatic, mild, moderate, severe, or critical. Ordinal logistic regression was used to examine the factors associated with COVID-19 severity. Vaccination was significantly associated with reduced severity of SARS-CoV-2 infection (OR: 0.044, 95% CI: 0.004–0.544, p=0.015). The use of prednisone at a dose >10 mg/d in the past 3 months did not significantly influence the severity of infection (OR: 4.198, 95% CI: 0.857–20.563, p=0.077).

## 4 Discussion

COVID-19 has significantly affected BP management, affecting medication regimens, appointment scheduling, and patient adherence to treatments. Some patients may experience a resurgence of BP symptoms due to therapy modifications or discontinuations prompted by the pandemic, along with potential side effects from SARS-CoV-2 infection or vaccination (7–9). These challenges may contribute to a decline in quality of life. Therefore, assessing the bidirectional effects of BP and COVID-19 is crucial for optimizing patient care. This cross-sectional survey aimed to investigate the impact of COVID-19 on BP patients, outlining their demographic and clinical profiles, COVID-19-related characteristics, and identifying factors influencing the risk and severity of SARS-CoV-2 infection in BP patients.

Our findings indicate that vaccines play a pivotal role in protecting BP patients against SARS-CoV-2 infection and severe COVID-19, aligning with previous studies (3, 10). Although underlying diseases and immunosuppressive medications may reduce vaccine effectiveness, prior research has consistently demonstrated that vaccination provides significant protection against severe COVID-19 cases (7, 11). Some studies have reported that vaccines may trigger new-onset AIBDs or exacerbate existing cases (7, 8, 12). Concerns about BP recurrence following vaccination emerged as the primary reason for vaccine hesitancy in our study. Contrary to these concerns, most patients in our study did not experience BP recurrence. Even those with disease exacerbation were managed effectively without significant consequences. This observation is consistent with the findings of Kasperskiewicz et al., who reported that BP occurrences after vaccination are rare and manageable (13). Thus, it is recommended that all immunocompromised dermatology patients receive the SARS-CoV-2 vaccine. To enhance efficacy and reduce the risk of disease flare-ups following vaccination, it is preferable to administer vaccines during the remission phase of the disease (14–16).

The mechanism underlying the relationship between COVID-19 vaccination and BP remains complex. Several potential mechanisms may contribute to BP onset following vaccination, including molecular mimicry, epitope spreading, bystander activation of self-reactive lymphocytes, polyclonal activation due to adjuvant reactions, and somatic mutation of immunoglobulin variable genes (17). However, Kasperkiewicz et al. demonstrated that circulating anti-SARS-CoV-2 antibodies do not cross-react with key autoantigens of AIBDs, including BP180 and BP230 (18). Additionally, Curman et al. suggest that COVID-19 vaccines not only prevent severe outcomes associated with infection but also reduce the risk of subsequent autoimmune complications (9). This dual role of COVID-19, both as an autoimmune trigger through infection and as a mitigator via vaccination, underscores the intricate interplay between infectious agents and immune regulation.

Our findings suggest that the use of prednisone at a dose >10 mg/d in the last 3 months may increase the risk of SARS-CoV-2 infection in BP patients. The relationship between glucocorticoids and SARS-CoV-2 infection risk remains contentious, with previous studies reporting conflicting results. Feng et al. reported that low-to-medium doses of glucocorticoids did not increase the risk of SARS-CoV-2 infection in pemphigus patients (19). However, their study did not include BP patients, whereas our research specifically focuses on this population. In contrast, Mahmoudi et al. identified the use of prednisone at a dose >10 mg/d in the last 3 months as an independent risk factor for SARS-CoV-2 infection in patients with AIBDs (20). Similarly, a study on another type of autoimmune disease, chronic inflammatory arthritis, showed that even low doses of prednisone were associated with higher rates of SARS-CoV-2 infection (21). The debate over the relationship between glucocorticoids and infection risk extends beyond COVID-19. While randomized controlled trials suggest that glucocorticoid therapy does not increase infection risk, observational studies consistently demonstrate an elevated risk of infections, including serious and opportunistic infections (22, 23). Furthermore, baseline glucocorticoid use has been linked to a dose-dependent increase in infection rates, with higher doses conferring greater risk (24).

Our findings also indicate that the use of prednisone at a dose >10 mg/d in the last 3 months does not appear to influence the severity of COVID-19. Conflicting results have been reported in this regard as well. Some studies found that systemic corticosteroids and adjuvant immunosuppressive agents did not affect infection severity or increase the risk of poor outcome (5, 25). Conversely, other studies identified prednisone doses >10 mg/d in the last 3 months as a factor that worsens COVID-19 severity (3, 10, 20). Zhang et al. observed that, in pemphigus patients, those receiving an oral corticosteroid dose ≥15 mg/d were more prone to COVID-19 aggravation, while no such association was identified in the pemphigoid group (26).

The varying effects of glucocorticoids and immunosuppressants on the risk and severity of SARS-CoV-2 infections may arise from the distinct roles these agents play during the triphasic course of COVID-19 (27). In the early phase, where the host immune response is crucial for controlling viral replication, immunosuppressive drugs may be detrimental. Conversely, in the advanced severe phase, these agents may offer protective benefits by mitigating excessive immune responses, such as secondary hemophagocytic lymphohistiocytosis, which can lead to acute respiratory distress syndrome, multiorgan failure, and mortality (5). These findings underscore the intricate interplay between autoimmune disease management, immunosuppressive therapy, and the pathophysiology of viral infections. Further studies with larger sample sizes are needed to clarify the precise role of immunosuppressive agents in COVID-19 progression.

While previous studies have identified age and comorbidities as risk factors for severe COVID-19 outcomes (3, 26, 28, 29), these factors did not achieve statistical significance in our study. This discrepancy may be attributed to the higher proportion of elderly patients in the BP cohort, many of whom have multiple underlying conditions, potentially introducing bias into the results (3). Additionally, our study categorized patients solely based on the presence of comorbidities, without detailed analysis of specific conditions, which may have obscured the varying impacts of individual comorbidities. In addition, a small sample size may also be a reason. Given these considerations, we recommend that elderly BP patients take additional precautions to prevent infection, adhere rigorously to social distancing measures, and follow established public health guidelines to minimize their risk.

Fever, cough, fatigue, and muscle soreness emerged as the most common symptoms in our cohort, consistent with previous research (19, 26). Gastrointestinal (GI) symptoms were also common in COVID-19-positive patients. Among these, gastrointestinal bleeding (GIB) demands special attention, as COVID-19 patients with GIB were more prone to death than non-GIB COVID-19 patients (30, 31). Coagulopathies, adoption of anti-coagulant therapies, and other viral effects make COVID-19 patients more prone to developing GIB. For BP patients, this risk is further compounded by two factors. First, glucocorticoid therapy may exacerbate gastrointestinal mucosal injury. Second, BP itself can involve mucosal erosions. Moreover, we propose future research could investigate whether BP patients experience excessive or prolonged bleeding from BP lesions, as this could provide a deeper understanding of the potential impact of COVID-19 on skin fragility and coagulation function.

The COVID-19 pandemic has underscored the increasing importance of telemedicine, particularly in visual specialties such as dermatology. During the pandemic, patients may delay or avoid seeking medical care, which can lead to adverse outcomes. Telemedicine offers clear advantages in addressing these challenges, providing a safe and effective method for ongoing medical follow-up while maintaining social distancing. Studies have demonstrated that teledermatology is as effective as in-person management for AIBDs (32). By leveraging online platforms, healthcare providers can communicate with patients, monitor changes in their conditions, and adjust treatment plans as necessary (33). It is recommended that patients with mild or stable BP use teledermatology for follow-up appointments (34). Hospitals should actively promote online consultations and facilitate convenient medication delivery services for patients.

Our study did not include a healthy control group. The absence of a control group made it challenging for us to evaluate the differences between BP patients and healthy individuals in terms of the infection rate, hospitalization rate, fatality rate, and the severity of COVID-19. In this study, we analyzed the impact of age, gender, BP duration, comorbidities, glucocorticoid use, and COVID-19 vaccination status on the risk and severity of SARS-CoV-2 infection. Owing to the lack of a healthy control group, we are unable to determine whether the research results are specific to BP patients or whether these results differ from those in the general population. Considering that BP is an autoimmune disease, it may influence the body’s immune response to COVID-19 infection.

This study has several limitations. First, the sample size was limited, and there was no inclusion of a healthy control group. Second, it was a single-center study, and data collection relied primarily on self-reported statements from patients. Third, the cross-sectional design limited causal inference. Fourth, unmeasured confounding factors, such as genetic predispositions, adherence to personal protective measures, and variations in SARS-CoV-2 strains, may have influenced the COVID-19 outcomes. Therefore, these results should be interpreted with caution. Future studies with longitudinal follow-up, larger sample sizes and more robust methodologies are necessary to confirm and validate these findings.

## 5 Conclusion

Our findings suggest that COVID-19 vaccination may reduce the risk of SARS-CoV-2 infection and protect against severe COVID-19. We recommend that BP patients receive the SARS-CoV-2 vaccine unless contraindicated. Additionally, we observed that the use of prednisone at a dose >10 mg/d in the last 3 months may have an increased risk of SARS-CoV-2 infection but does not appear to impact infection severity. Physicians should carefully weigh the risks and benefits when prescribing medications. Further research with larger sample sizes and comprehensive monitoring of BP patients is essential to enhance our understanding of the risks and impacts of SARS-CoV-2 infection in BP patients.

## Data Availability

The raw data supporting the conclusions of this article will be made available by the authors, without undue reservation.
